# A Masked Representation Alignment-Based Self-Supervised Learning Method for Radar Emitter Recognition

**DOI:** 10.3390/s26196114

**Published:** 2026-09-26

**Authors:** Yixin Zuo, Wenjuan Ren, Guangzuo Li

**Affiliations:** 1 Aerospace Information Research Institute, Chinese Academy of Sciences, Beijing 100094, China; renwj@aircas.ac.cn (W.R.); ligz@aircas.ac.cn (G.L.); 2 Research Department of Cyber-Electromagnetic Space Information Technology, Chinese Academy of Sciences, Beijing 100094, China

**Keywords:** self-supervised learning, radar emitter recognition, PDW, masked representation alignment

## Abstract

Radar emitter recognition based on pulse description words (PDWs) serves as a fundamental prerequisite for target identification and tracking in electronic support measures (ESM). Although self-supervised learning (SSL) has been widely applied to text and image tasks, few effective SSL paradigms are available for the feature representation of radar PDWs. In this paper, a novel masked representation alignment-based self-supervised learning (SSL-MRA) method is proposed for radar emitter feature learning and recognition. Firstly, a dual-branch Transformer encoder is designed to extract contextual representations from both masked and unmasked tokens. Secondly, a cross-attention Transformer-based predictor is constructed to recover the masked representations from unmasked features. Furthermore, a codebook-based tokenizer is developed to learn discrete representations of masked inputs. Based on the masked prediction mechanism, two pretext tasks are established for the pre-training of SSL-MRA. Specifically, a masked discrete representation alignment task is adopted to replace traditional reconstruction-based pre-training, which implements codebook classification for semantic discretization. Meanwhile, a masked prediction representation alignment task is constructed to constrain the high-dimensional semantic consistency of feature embeddings. Experimental results on both simulated and real measured datasets demonstrate that the proposed method achieves superior feature representation capability. It yields an average recognition accuracy of 94.95% on source-domain data, outperforming all baseline methods. Moreover, the proposed SSL-MRA also achieves better cross-domain transfer performance than the mainstream masked autoencoder method.

## 1. Introduction

Radar emitter recognition is a core intelligent perception technology in modern electronic support measures (ESM) systems, undertaking key tasks such as electromagnetic signal parsing, emitter attribute identification, and battlefield electromagnetic situation reconstruction [1,2,3]. As standardized quantitative description of radar pulse signals, radar pulse description words (PDWs) record critical pulse parameters. As shown in Figure 1, the PDW of each intercepted pulse is a multi-parameter sequence, including time of arrival (TOA), radio frequency (RF), pulse width (PW), pulse amplitude (PA), and direction of arrival (DOA). Typically, when processing PDW data, we consider only three types of parameters: TOA, RF, and PW. And we convert the TOA into pulse repetition interval (PRI) using first-order differencing in this paper. These multi-dimensional time-series parameters effectively characterize the operating mechanisms, functional attributes, and working states of radar emitters, serving as the dominant data source for radar signal sorting and intelligent recognition [4].

In complex practical electromagnetic battlefield environments, modern multifunctional radars exhibit agile parameter variations, overlapping parameter intervals, and diverse working modes [5]. Meanwhile, complex electromagnetic interference inevitably causes pulse loss, spurious pulse disturbances, and measurement noise in acquired PDW data. These non-ideal factors severely degrade the effective feature distribution of pulse sequences, resulting in low feature discriminability among homogeneous radars and poor robustness of recognition algorithms, which poses substantial challenges for accurate and reliable radar emitter recognition.

With the rapid advancement of deep learning, data-driven radar emitter recognition methods have gradually replaced traditional manual feature extraction and statistical classification algorithms and become the mainstream technical paradigm in this field. Existing intelligent recognition methods [6,7,8] are predominantly built upon supervised learning frameworks, which rely on large-scale labeled PDW datasets to establish end-to-end feature mapping and classification models. Typical supervised networks, including CNN, LSTM, and Transformer, have been widely applied to radar signal feature mining and classification, achieving promising performance under ideal experimental conditions. Nevertheless, practical engineering applications reveal two critical limitations of supervised radar recognition methods. On the one hand, most models are trained and validated based on artificially simulated PDW data. Ideal simulation environments cannot fully reproduce the noise distortion, missing pulses, and complex interference characteristics of real measured data, leading to severe domain mismatch between experimental models and practical scenarios [9]. On the other hand, supervised learning models are task-driven and label-dependent with fixed feature learning paradigms. Such models lack adaptive generalization capability for unknown radar types and time-varying electromagnetic environments, inevitably suffering severe performance degradation across different datasets and application scenarios [10].

Self-supervised learning (SSL) enables the learning of generalizable feature representations by constructing effective pretext tasks from inherent data characteristics without relying on task-specific annotations. Therefore, SSL trained on massive unlabeled data has been widely deployed in natural language processing (NLP) and computer vision (CV). In particular, the Masked Autoencoder (MAE) proposed by He et al. [11], built upon the ViT architecture [12] architecture, achieves outstanding fine-tuning and transfer learning performance on the ImageNet dataset. Accordingly, masked modeling techniques [13,14,15] have been gradually introduced into time-series signal analysis tasks, including industrial condition monitoring and physiological signal processing [16]. Recently, researchers have migrated SSL methods from physiological signal analysis to radar signal processing for radar modulation recognition [17,18] and working mode recognition [10]. Most existing studies focus on original I/Q signals, which contain long-duration waveforms, explicit modulation patterns, and periodic characteristics. In contrast, research on SSL-based PDW sequence recognition remains insufficient. Different from conventional I/Q time-series data, radar PDW sequences exhibit shorter temporal lengths, structured parameter distributions, multi-parameter coupling characteristics, and high noise sensitivity. Directly adopting general time-series SSL models for PDW feature learning leads to insufficient feature pertinence, poor robustness, and limited performance improvement in downstream recognition tasks [19].

In view of the above research gaps, this paper proposes a masked representation alignment-based self-supervised learning (SSL-MRA) method for radar emitter recognition. Unlike existing supervised radar recognition schemes and general time-series SSL frameworks, the proposed method designs a dual-branch Transformer encoder to synchronously extract contextual features from both masked and unmasked PDW sequences. Combined with cross-attention Transformer-based masked prediction representation alignment and masked discrete representation alignment pretext tasks, the SSL-MRA method enables fine-grained and robust feature learning for radar pulse sequences. Extensive experiments on both simulated and real measured datasets verify that the proposed method achieves superior feature representation capability, better fine-tuning accuracy, and stronger cross-scene generalization than baseline methods. The main contributions of this paper are summarized as follows:

(1) A dual-branch Transformer encoder architecture is designed for PDW sequence feature extraction, which compensates for incomplete feature mining defects existing in conventional single-branch time-series modeling.
(2) A multi-dimensional self-supervised pretext task integrating cross-attention prediction and discrete representation alignment is proposed to enhance the discriminability and robustness of PDW feature representations.
(3) The effectiveness and practical superiority of the proposed method are comprehensively validated on both simulated and real measured data, providing a new feasible solution for robust and generalized radar emitter recognition in complex electromagnetic environments.

### 1.1. Supervised Learning for Radar Emitter Recognition

Supervised deep learning has long served as the dominant technical paradigm for radar emitter recognition. Benefiting from the powerful end-to-end feature learning capacity of deep neural networks, classic supervised architectures including Convolutional Neural Network (CNN), Long Short-Term Memory (LSTM), and Transformer have been extensively deployed for PDW sequence feature extraction and radar recognition tasks [4]. Traditional radar recognition approaches rely on manual feature engineering, which demands specialized domain expertise to extract statistical and transform-domain pulse features, suffering from low feature utilization efficiency and weak environmental adaptability. By contrast, data-driven supervised learning frameworks [6,7,8] can automatically mine latent feature patterns from PDW time-series data, effectively circumventing the drawbacks of handcrafted feature design. In terms of spatial feature extraction, CNN-based models are widely adopted to capture local correlation characteristics of radar pulse data. Researchers convert one-dimensional PDW sequences or original radar I/Q signals into two-dimensional feature maps, and they then employ convolution kernels to excavate local spatial distributions of pulse parameters, yielding stable recognition accuracy under conventional single-scene conditions [6]. For time-dependent pulse sequences, LSTM and Gated Recurrent Unit (GRU) networks are leveraged to model temporal dependencies embedded in PDW data, which accurately capture time-varying patterns of RF, PW and PRI parameters and compensate for the limited temporal perception of convolutional models [7]. With advances in sequence modeling, Transformer architectures equipped with multi-head self-attention have been introduced into radar recognition tasks. Such models are capable of capturing global long-range dependencies across pulse sequences and further strengthen the feature representation capacity of long-duration PDW data [8].

Currently, abundant research has greatly advanced the development of supervised radar emitter recognition. Fan [5] constructed a novel feature extraction and clustering framework oriented to multifunctional radar working mode identification, enabling adaptive mining of multi-scale features corresponding to radar working states. Yang [9] integrated deep metric autoencoders into radar open-set recognition, mitigating the challenge of unknown emitter classification to a certain degree. Liu [10] further introduced incremental learning to radar recognition pipelines, supporting continuous learning of unseen radar categories while alleviating catastrophic forgetting. Wang [4] generated 2D feature maps from the distribution properties of pulse sequences and fused local and global segmentation information for classification, achieving state-of-the-art performance on a dataset covering eight types of radar emitters. Despite outstanding classification accuracy achieved on fixed experimental datasets, supervised learning methods suffer from inherent, hard-to-mitigate limitations in practical engineering deployments. First, nearly all supervised models require labeled samples for training. Annotating radar PDW data consume substantial professional labor and time, leading to a severe shortage of labeled samples in real-world electromagnetic scenarios [20]. Second, supervised models exhibit strong dataset bias; the feature patterns learned within a specific dataset cannot be readily transferred to unseen electromagnetic environments or novel radar types, resulting in poor generalization performance. Third, most training datasets adopted in the existing literature consist of idealized simulated data, which fail to replicate complex noise interference and pulse distortion encountered in field measurements, ultimately degrading the practical robustness of deployed models.

### 1.2. General Time-Series Self-Supervised Pre-Training Methods

Driven by the remarkable feature representation performance of Transformer [21], BERT [22,23], ViT [24], and MoCo [25], a spectrum of classic self-supervised learning (SSL) paradigms has been proposed for natural language processing (NLP) and computer vision (CV), including masked modeling [11,13,14,15], contrastive learning (CL) [26,27,28,29,30], and autoregressive prediction [31,32,33].

Masked modeling, represented by the masked autoencoder (MAE) [11], implements unsupervised learning by randomly masking partial data tokens and reconstructing the missing information. This mechanism effectively excavates local and global structural correlations within time-series data and yields robust feature embeddings. For example, Cheng et al. [13] devised a decoupled masked autoencoder for time-series representation based on MAE, and validated its superior feature learning and transfer capabilities on multiple public benchmarks in 2022. In 2024, Wang [15] presented a lightweight SSL framework customized for multi-lead ECG signals. The framework optimizes segment-wise masked pre-training via fluctuated reconstruction targets and layered regularization strategies, effectively alleviating data redundancy and overfitting risks for ECG recordings. Contrastive learning [29] generates positive and negative sample pairs through data augmentation, and learns discriminative embeddings by minimizing feature distances between positive pairs while maximizing distances between negative pairs. This paradigm demonstrates prominent advantages in small-sample and cross-domain learning tasks. Hu [14] combined MAE and contrastive learning to build a spatio-temporal representation alignment framework for EEG sequence classification. Autoregressive SSL models adopt sequential prediction as the pretext task to simulate temporal evolution patterns of time-series signals, making them well-suited for modeling sequences with long-range dependencies [33]. Wang et al. [31] proposed TimeDART, a unified self-supervised pre-training framework for time series. It integrates autoregressive Transformer modeling and patch-level denoising to capture both long-term temporal trends and subtle local variations. Extensive forecasting and classification experiments on multiple public datasets verify that TimeDART outperforms mainstream SSL baselines. Liu et al. [32] put forward a decoder-only large time-series foundation model, which unifies forecasting, missing value imputation, and anomaly detection into autoregressive generative tasks and achieves competitive cross-domain generalization performance. Although representative works such as TimeMAE, EEGPT, TimeDART, and Timer attain promising results on general sequential datasets, few of them introduce targeted optimizations to accommodate the unique properties of radar PDW time series, including multi-parameter coupling and severe noise contamination.

### 1.3. Self-Supervised Pre-Training Methods for Radar Signal

As self-supervised learning has achieved extraordinary success in generic time-series modeling, researchers have begun to explore SSL applications in radar signal processing, covering I/Q signal representation learning, modulation recognition, and radar emitter recognition. Such methods leverage massive unlabeled radar data for model pre-training, aiming to address the scarcity of annotated samples and weak generalization plaguing traditional supervised algorithms.

For instance, Wang [18] proposed a diffusion-based self-supervised pre-training scheme for original radar I/Q signals, which enables unsupervised feature learning for microwave waveforms and improves model adaptability to signal amplitude and phase fluctuations. Liu [17] adopted contrastive SSL to tackle radar modulation recognition tasks, significantly boosting the anti-interference capacity of models under complex electromagnetic clutter. Zhou [34] pioneered the application of autoregressive SSL to radar modulation identification, realizing effective representation learning for I/Q data and opening a new research direction for SSL-driven intelligent radar signal processing. Zhang et al. [19] further developed feature-aligned self-supervised learning for radar open-set recognition, enhancing the model’s ability to distinguish unseen radar emitters. Ren et al. [35] proposed GAE, an improved MAE pre-training architecture tailored to PDW data. The method converts PDW sequences into two-dimensional images for training, and experimental results confirm that GAE delivers stronger radar emitter recognition performance than vanilla MAE. Nevertheless, most existing SSL solutions for radar signals have two obvious limitations. First, the majority of studies focus on low-level I/Q waveforms or simply convert PDW sequences into static images. Second, these works rely on a single pretext task for optimization. Such designs fail to extract robust discriminative features from noisy, multi-parameter coupled PDW sequences collected in complex electromagnetic environments [36,37].

## 2. Materials and Methods

### 2.1. Problem Description

Radar emitter recognition relying on pulse description word (PDW) sequences faces great practical challenges in complex electromagnetic battlefield environments, including measurement noise, pulse loss, spurious pulses, and severe feature overlap among different emitters. Supervised recognition approaches are heavily dependent on high quality labeled PDW samples and suffer from poor generalization when transferring from simulated datasets to real measured data.

Self-supervised learning provides a feasible way to learn discriminative representations from massive unlabeled PDW time-series data. Nevertheless, existing self-supervised paradigms adapted for radar PDW sequences still exhibit prominent technical limitations. First, mainstream latent target prediction methods like masked autoencoder (MAE) mainly optimize token-level signal reconstruction. They lack explicit feature alignment constraints for incomplete masked PDW segments, which degrades feature discriminability when multiple emitters have highly overlapped PDW parameter distributions and low feature separability. Typical codebook-based approaches focus on discrete token reconstruction over original inputs, performing feature learning by mapping data to discrete vectors. In addition, conventional contrastive learning frameworks require constructing large numbers of negative sample pairs for optimization, which introduces extra computational overhead and cannot well match the multi-parameter coupling characteristics of radar pulse streams. Currently, few methods exist to consider joint constraints over both continuous latent features and discrete semantic patterns. Consequently, the learned embeddings are prone to overfitting the measurement noise specific to the dataset; their performance degrades significantly in complex electromagnetic environments characterized by measurement noise, pulse loss, and spurious pulses. By contrast, our SSL-MRA performs dual-branch positive-only feature matching, jointly optimizing continuous latent consistency and discrete semantic discrimination on masked incomplete PDW embeddings, rather than reconstructing original PDW signals or discrete input tokens. We highlight that our momentum-updated asymmetric dual-branch design eliminates the requirement for large-scale negative sample pairs in conventional contrastive learning, and the vector-quantized (VQ) pretext goal acts as semantic constraint for latent representations instead of reconstructing input-side tokens.

To mitigate the above drawbacks, this paper proposes a masked representation alignment-based self-supervised learning (SSL-MRA) framework for radar emitter recognition. Different from reconstruction-only masked models and negative pair-dependent contrastive schemes, the proposed method builds an asymmetric momentum updated dual branch structure to implement positive-only feature matching. It jointly optimizes continuous feature consistency and discrete semantic discrimination via two pretext tasks, enhancing embedding quality for incomplete masked PDW sequences. The framework obtains more robust representations and higher recognition performance under both complex environmental interference and low separability of radar PDWs with similar parameters. The overall architecture of SSL-MRA is illustrated in Figure 2. First, a preprocessing and embedding module converts the input multi-parameter PDW sequences into masked and visible high-dimensional embeddings. Next, a dual-branch contextual feature representation module learns feature representations for the two groups of embeddings. Finally, the masked prediction representation module and masked discrete representation module jointly act as dual pretext tasks to optimize the self-supervised model.

### 2.2. Data Preprocessing and Embedding

Restricted by inconsistent receiving hardware, complex electromagnetic environments and diverse pulse sorting algorithms, original measured radar PDW sequences suffer from uneven sequence lengths and inconsistent feature dimensions. Hence, all PDW samples require unified standardization and normalization before network input. In this work, cyclic padding and cropping operations are adopted to unify variable-length PDW sequences into a fixed dimension of 64∗3, where 64 denotes the sequence length and 3 stands for the number of pulse parameters. The three dimensions correspond to the Pulse Repetition Interval (PRI), Radio Frequency (RF), and Pulse Width (PW), respectively. PRI and PW are uniformly converted to microseconds (μs), while RF is scaled to megahertz (MHz). Afterwards, a hybrid normalization strategy combining Min–Max scaling and independent dimension normalization is utilized to map all three parameter dimensions into the range [0,1].

In the embedding stage, sliding window segmentation is applied to split each 64-length standardized sequence into non-overlapping 4∗1 patches. A one-dimensional convolutional layer is then used to extract cross-channel local embeddings for each patch. Afterwards, a random masking strategy with a masking ratio of 50% is implemented to generate visible unmasked embeddings $x_{\bar{M}}$ and masked embeddings $x_{M}$. Under sufficient training iterations, this design guarantees that each patch shares an equal probability of being masked.

### 2.3. Feature Representation Learning

#### 2.3.1. Masked Prediction Feature Alignment

Typically, self-supervised learning based on a masked model will introduce additional masked information into the embedding computation during the pre-training stage. However, the training data used in the fine-tuning stage lack a masking strategy, and the optimization objectives differ between the two stages, leading to inconsistent learning goals at different stages. Thus, we employ a dual-branch vanilla Transformer encoder module to separately learn masked feature representations $z_{M}$ and visible feature representations $z_{\bar{M}}$.

(1) $$z_{\bar{M}}=Enc\left(x_{\bar{M}}+pos\left(x_{\bar{M}}\right)\right)$$

(2) $$z_{M}=MEnc\left(x_{M}+pos\left(x_{M}\right)\right)$$

where $x_{M}$ and $x_{\bar{M}}$ denote masked embeddings and unmasked embeddings (visible embeddings), pos(·) stands for positional encoding, and MEnc(·) refers to the momentum encoder.

The core insight of representation learning lies in the fact that latent spaces capturing intrinsic data distributions possess far lower dimensionality than original input spaces. Latent embeddings can highlight meaningful temporal patterns embedded in time-series inputs while suppressing irrelevant measurement noise. To realize this property, leveraging the consistency of deep feature space representations to construct pretext tasks for self-supervised learning is a significant approach. Thus, we predict new feature representation of masked region based on the feature representation from visible parts learned by the encoder, while ensuring that the predicted masked features remain consistent with the original masked feature representations learned by the momentum encoder.

Specifically, a cross-attention Transformer module takes unmasked (visible) features and mask position information as inputs to reconstruct masked representations. We reinitialize a masked feature vector based on the masked position as the prediction target, and we then use it as the query of the cross-attention transformer. In addition, we construct the value and key from the feature representation $z_{\bar{M}}$ of the visible input part. Then, we can obtain the masked prediction feature representation ${\hat{z}}_{M}$ through the cross-attention transformer module. Through the above operations, we obtained masked feature representations from two different views, thus enabling feature representation alignment using contrastive learning. The core principle of contrastive learning is to narrow the embedding distance between positive sample pairs and enlarge the gap between negative pairs. Differently, we only align positive sample pairs constructed by the masked prediction feature representation and its original masked feature representation learned by momentum encoder without considering negative sample pairs. Since the masked feature representations from the momentum encoder and predictor are both continuous latent vectors, we use mean squared error (MSE) loss to form the feature prediction alignment optimization objective $L_{mpa}$ shown in Equation (4).

(3) $${\hat{z}}_{M}=Pred\left(z_{\bar{M}}\right)$$

(4) $$L_{mpa}=\frac{1}{N}\sum_{j=1}^{N}{∥{\hat{z}}_{M}^{j}-z_{M}^{j}∥}_{2}^{2}$$

where Pred(·) denotes the stacked cross-attention and feed-forward layers. ${\hat{z}}_{M}^{j}$ is the predicted masked feature from the predictor, and $z_{M}^{j}$ is the original masked feature extracted by the momentum encoder.

We implement a gradient-stop strategy on the momentum encoder, whose weights are updated via a moving average (MA) rule throughout pre-training. The detailed update rules for the momentum encoder and encoder are formulated as follows:

(5) $$W_{MEnc}\leftarrow \eta \cdot W_{MEnc}+\left(1-\eta \right)\cdot W_{Enc}$$

(6) $$W_{Enc}\leftarrow W_{Enc}-\gamma \cdot \nabla_{Enc}L_{mpa}$$

where η∈[0,1] is a momentum coefficient for the momentum-based moving average. With a large value of η, the momentum encoder slowly approximates the encoder. For the proposed method, we find η=0.99 performs effectively. Further, ∇ is the gradient and γ denotes the learning rate for stochastic optimization. As shown in Equation (6), the direction of updating MEnc completely differs from that of updating $W_{Enc}$. Finally, MEnc converges to equilibrium by the slow-moving average. At each training iteration, only the encoder and the predictor receive gradient updates derived from alignment loss.

During fine-tuning, only the vanilla Transformer encoder is retained to extract feature representations from complete PDW sub-sequences, whose outputs are fed into the classification head for downstream emitter recognition. The momentum encoder is discarded at this stage, ensuring masked representations are decoupled from unmasked information for stable inference.

#### 2.3.2. Masked Discrete Feature Alignment

Conventional masked self-supervised learning (SSL) paradigms solely rely on reconstruction loss as their core optimization objective. Under this training target, the encoder is forced to prioritize pixel-level or token-level recovery of original input PDW sequences, and the learned feature representations tend to overfit trivial noise and measurement interference unique to the training dataset rather than extracting discriminative semantic patterns related to radar emitter categories. When transferred to downstream radar recognition tasks, such noise-biased embeddings often converge to suboptimal solutions and degrade the model’s classification accuracy, especially on real measured PDW data contaminated by pulse loss and complex electromagnetic clutter.

To address this inherent limitation of single reconstruction loss, we draw inspiration from vector quantization (VQ) theory proposed in [38] and designed an end-to-end tokenizer module. This module bridges continuous latent feature space and discrete semantic space, converting dense feature representations extracted from masked PDW sub-sequences into sparse, interpretable discrete codewords without introducing extra offline clustering steps. The core of the tokenizer is a codebook embedding matrix $E=\left\{e_{1},e_{2},e_{3},\ldots ,e_{K}\right\}\in R^{K*d}$, which stores *K* latent prototype vectors with dimension *d* identical to the embedding size of PDW sub-sequences.

During the pre-training forward propagation, two groups of masked feature vectors are simultaneously fed into the tokenizer: the original masked embedding $x_{M}$ generated from the preprocessing module (serving as the discrete target), and the predicted masked representation ${\hat{z}}_{M}$ output by the cross-attention predictor branch (serving as the prediction). It should be emphasized that the tokenizer operates on the encoder-input-level $x_{M}$, not on the output of the momentum encoder; the momentum encoder is only used in the MPA branch for continuous feature alignment. For each input sub-sequence vector, we calculate its similarity to every prototype vector in the codebook via cosine similarity, a metric insensitive to vector magnitude and suitable for measuring the matching degree of temporal feature distribution of multi-parameter PDW data. Each masked embedding $x_{M}$ and predicted masked representation ${\hat{z}}_{M}$ are then, respectively, mapped to the codeword with the highest similarity score, and the corresponding serial number of this optimal codeword are recorded as the token index $y_{t}$ and $y_{p}$. $y_{p}$ can be expressed as the maximum cosine similarity between ${\hat{z}}_{M}$ and the codebook vectors as shown in Equations (7) and (8). Similarly, $y_{t}$ is the maximum cosine similarity between $x_{M}$ and the codebook vectors.

(7) $$y_{p}=\underset{k}{\arg\;\max}\;l_{k}\;k\in \left[1,K\right]$$

(8) $$l_{k}=\frac{{\hat{z}}_{M}\cdot e_{k}}{∥{\hat{z}}_{M}∥\;e_{k}∥},\;k=1,2,\ldots ,K$$

where $l_{k}$ denotes the cosine-similarity logit between predictor output embedding ${\hat{z}}_{M}$ and each codebook prototype vector $e_{k}$, and *K* is the total codebook size.

Softmax with temperature τ converts logits $\left\{l_{k}\right\}$ into predicted categorical probability distribution $\left\{p_{k}\right\}$ as shown in Equation (9). Thus, the probability of predicted prototype index $y_{p}$ can be expressed as $p_{y_{p}}$, and the probability of target distribution from the original masked input prototype index $y_{t}$ can be expressed as $p_{y_{t}}$.

(9) $$p_{k}=\frac{\exp\left(l_{k}/\tau \right)}{\sum_{i=1}^{K}\exp\left(l_{i}/\tau \right)}$$

Each discrete codeword corresponds to a typical temporal pattern of PDW sub-sequences formed by the coupling of PRI, RF, and PW multi-dimensional pulse parameters. Therefore, the matched token indices naturally serve as intrinsic self-supervised supervision signals that reflect the inherent structural characteristics of masked PDW segments. On this basis, we construct the masked discrete representation alignment optimization objective $l_{mda}$, as formulated in Equation (10). This loss adopts cross-entropy to constrain the token indices assigned to the predicted representation ${\hat{z}}_{M}$ and the original masked embedding $x_{M}$ to be consistent. By minimizing the cross-entropy loss between two sets of token labels, the model is guided to learn discrete embeddings with strong category discrimination, which complements the continuous feature alignment loss of the prediction branch and further improves the robustness of PDW feature representation under noisy real measurement environments. Following the self-distillation paradigm, a stop-gradient is applied to the target-side token distribution $p_{y_{t}}$, and only the prediction-side distribution $p_{y_{p}}$ receives gradients. This prevents representational collapse by ensuring the target remains a stable objective that the predictor must learn to match.

(10) $$l_{mda}=CE\left(y_{t},p_{y_{p}}\right)=-\sum_{i=1}^{N}y_{t}^{i}*\log p_{y_{p}^{i}}$$

During the forward pass, the index of the nearest embedding is assigned as the current embedding vector’s discrete codeword. During the backward pass, the stop-gradient operation takes effect, and the non-differentiable argmax mapping is resolved via the straight-through estimator (STE) [39]. The gradient bypasses the codebook vectors and is propagated directly to the masked feature vectors prior to discrete assignment, meaning the non-differentiability issue is resolved. In addition, the codebook is updated exclusively via an exponential moving average (EMA) algorithm without gradient backpropagation, as formulated in the Equations (11a–c). At training step *t*, each prototype $e_{i}$ is updated by weighting the $n_{i}$ closest input vectors ${\hat{z}}_{M}^{i}$ at the current step and its historical value from step t−1. $N_{i}$ counts the total number of patches assigned to prototype *i*, $m_{i}$ accumulates weighted feature sums, and ζ is the EMA discount factor.

(11a) $$N_{i}^{t}=N_{i}^{t-1}*\zeta +n_{i}*\left(1-\zeta \right)$$

(11b) $$m_{i}^{t}=m_{i}^{t-1}*\zeta +\sum_{j}^{n_{i}}{\hat{z}}_{M}^{i}*\left(1-\zeta \right)$$

(11c) $$e_{i}=\frac{m_{i}^{t}}{N_{i}^{t}}$$

### 2.4. Model Optimization Objectives

In the pre-training phase, the model is jointly optimized with mask prediction feature alignment loss and masked discrete feature alignment loss. The overall pre-training loss $l_{pretrain}$ is defined as follows:

(12) $$l_{pretrain}=l_{mpa}+\alpha l_{mda}$$

where α is a hyperparameter to balance the two loss terms.

In the fine-tuning stage, we directly input the embedding vectors of all the radar PDW sub-sequences into the vanilla Transformer encoder, and then followed a prediction head to achieve the recognition of radar emitter. As shown in Equations (13)–(15), we utilize the cross-entropy loss between the radar type labels and predict results to optimize the parameters from encoder and prediction layer.

(13) $$z_{M\cup \bar{M}}=Enc\left(x+pos\left(x\right)\right)$$

(14) $$l_{finetune}=-\frac{1}{N}\sum_{i=1}^{N}\sum_{m=1}^{M}y_{m}^{i}\;\log p_{m}^{i}$$

(15) $$p_{m}^{i}=\frac{\exp\;(\mathrm{CLS}(z_{M\cup \bar{M}}^{i})}{\sum_{j=1}^{M}\exp\;\left(\mathrm{CLS}{\left(z_{M\cup \bar{M}}^{i}\right)}_{j}\right)}$$

where $y_{m}^{i}$ is the ground-truth emitter category label *m* of the *i*th sample, $p_{m}^{i}$ represents the probability that the *i*th sample is predicted as category *m*, and CLS(·) represents the classification prediction head.

### 2.5. Dataset Description and Implementation Details

To evaluate the cross-domain transferability of the proposed SSL-MRA, this paper conducts comprehensive experiments on four benchmark datasets for pre-training and downstream recognition task fine-tuning. These include one simulated dataset SD5 and three real measurement datasets RD13, RD14, and RD11.

(1) Simulated Data (SD5): A simulated radar PDW dataset synthesized with measured noise, pulse lost, and spurious pulse. It contains 5 radar types, with each radar type containing 20,000 PDWs samples, and each sample has a uniform length of 64.
(2) Real Data (RD13): A real measured radar PDW dataset acquired via a single-satellite localization system. It contains 13 shipborne radar types, totaling 23,644 samples, with lengths ranging from 33 to 200, covering the L-band, C-band, S-band, and X-band.
(3) Real Data (RD14): A real measured radar PDW dataset acquired via a single-satellite localization system. It contains 14 land-based radar types, totaling 19,799 samples, with lengths ranging from 33 to 200, L-band, C-band, and S-band.
(4) Real Data (RD11): A real measured radar PDW dataset acquired via a multi-satellite time difference of arrival localization system. It contains 11 shipborne radar types, totaling 12,569 samples, with lengths ranging from 15 to 1094, L-band, C-band, and S-band.

The PDW sequences in RD13, RD14, and RD11 were acquired by single-satellite or multi-satellite TDOA localization systems. The ground-truth radar-type labels were assigned by domain experts (radar intelligence analysts) through a manual annotation procedure. Specifically, each intercepted PDW sequence was individually inspected by experienced analysts, who determined the radar emitter type by jointly considering four sources of information: (i) the parameter fingerprints extracted from the PDW sequence, including the radio frequency (RF), pulse width (PW), and pulse repetition interval (PRI); (ii) a curated intelligence database containing known emitter parameters, platform associations, and deployment records; (iii) a knowledge base of radar operating modes and signal characteristics; and (iv) the analysts’ domain expertise accumulated from long-term electromagnetic signal analysis. To reduce annotation bias and label noise, each sample was independently annotated by at least two analysts, and disagreements were resolved through discussion and consensus; samples that could not be confidently labeled were excluded from the final datasets. We acknowledge that, as with any real measured datasets, a small degree of label noise may remain due to the inherent ambiguity of electromagnetic signal interpretation; however, the expert-annotated labels are considered reliable and serve as the ground truth for both training and evaluation.

Four distinct datasets are utilized for pre-training and fine-tuning. The dataset partitioning is summarized in Table 1. For the simulated dataset SD5, 80,000 samples were allocated for pre-training, while the remaining 20,000 samples were used for supervised fine-tuning in the radar recognition task, split into training and testing sets at a 4:1 ratio. Regarding the three categories of real measured data, we designated 250 samples from each radar class for fine-tuning, while using all remaining samples for pre-training. Within the fine-tuning set, 50 samples from each category were further set aside to serve as the test set. To prevent data leakage, all data splitting is performed at the pulse-stream level. Each original PDW recording is treated as an independent unit, and all segments cropped from the same recording are assigned to the same subset. No recording is shared across the pre-training, fine-tuning (train), and fine-tuning (test).

The input length of radar PDW is 64, the patch size is 4, and the masking ratio is set to 50%. The dual-path transformer encoder contains 8 attention heads and 16 layers with total 128 embedding dimensions. The predictor consists of 8 cross-attention layers, and the codebook’s size is set to 256. The hyperparameters for the masked prediction alignment loss and masked discrete alignment loss are set to 1 and 0.2, respectively. Both pre-training and fine-tuning employ the Adam optimizer with an initial learning rate of 1 × 10^−3^, and a step decay of learning rate is applied with a step size of 100 during fine-tuning. The experiments on all datasets involve 2000 pre-training epochs and 1000 fine-tuning epochs. Dropout is set to 0.2, the batch size during pre-training is 2048, and during fine-tuning it is 512. Xavier initialization is used for parameter initialization. All experiments are built and trained in the Pycharm compiler and pytorch deep learning environment on an Inspur server equipped with a single H100 80GB GPU and an i9-13900K CPU.

## 3. Results

We compare the proposed SSL-MRA method with mainstream contrastive self-supervised frameworks (MoCo [25]), three time-series self-supervised models (GAE [35], 1D-MAE [14], TimeDART [31]), as well as two classic supervised baselines (vanilla Transformer [21], ResNet-50 [40]). In addition, ablation experiments are conducted to evaluate the effectiveness of the two core components in our framework, namely the masked prediction alignment module and masked discrete alignment module.

- Resnet-50: A standard 50-layer convolutional neural network designed to extract spatial feature maps from transformed PDW sequences.
- Transformer: Basic self-attention architecture adopted as the supervised baseline, without customized time-series pretext tasks. Specifically, it employs a standard Transformer encoder with patch size = 1 (single-pulse tokenization, where each PDW pulse is treated as one token), a dropout rate of 0.1, and a fixed learning rate of 1 × 10^−3^ without learning rate decay.
- TimeDART: Autoregressive generative self-supervised framework dedicated to time-series pre-training.
- MoCo: Momentum-based contrastive learning paradigm for unsupervised discriminative feature learning.
- 1D-MAE: One-dimensional masked autoencoder specially optimized for sequential signal representation learning.
- GAE: Generalized autoencoder (GAE)-based multi-branch mask structures with Ulam stability for radar pdw representation learning.

### 3.1. Comparison Experiments

#### 3.1.1. Comparison of Source-Domain Recognition Performance

To quantitatively evaluate the performance of the proposed self-supervised learning framework for radar emitter recognition, we first report the recognition accuracy on the source domain of all datasets and conduct comprehensive comparisons with multiple mainstream deep learning baselines. We conducted multiple experiments across four datasets using five random seeds and calculated the mean and standard deviation. The recognition accuracy of our SSL-MRA and all competing methods on the downstream radar identification task is summarized in Table 2.

Overall, the proposed SSL-MRA achieves an average accuracy of 94.95% across the four datasets, surpassing all comparative approaches. For the simulated dataset SD5, the best-performing supervised baseline (Transformer) only attains 91.32%, whereas our SSL-MRA reaches 99.25%. This remarkable gain outperforms both self-supervised competitors (TimeDART, MoCo, 1D-MAE) and supervised models (Transformer, ResNet-50). For the three real measured datasets RD13, RD14 and RD11, the recognition accuracy of ResNet-50 and Transformer drops below 85%. TimeDART yields accuracy lower than 80% on all real measured datasets. MoCo achieves 90.42% accuracy on RD13, while 1D-MAE obtains 96.43%, 91.65%, and 83.16% on RD13, RD14, and RD11 respectively. However, GAE achieved higher recognition accuracy than 1D-MAE on both RD13 and RD14, even reaching an average accuracy of 95.94% on RD14, compared to 95.47% for the proposed method. Conversely, GAE’s accuracy on RD11 was only 73.96%, far below that of both 1D-MAE and our method. Notably, our SSL-MRA hits 86.68% on RD11, nearly 4 percentage points higher than 1D-MAE. Furthermore, SSL-MRA attains highest accuracy on both SD5 and RD13 among all evaluated methods, and it achieves an average accuracy of 94.95% across the four datasets, which is the highest.

#### 3.1.2. Comparison of Cross-Domain Generalization Performance

To evaluate the cross-domain generalization capability of the proposed SSL-MRA, we perform pre-training on each of the four datasets individually and test the recognition performance on cross target domains under different transfer settings. We further benchmark our approach against 1D-MAE and GAE as the primary comparison baseline. It should be noted that the cross-domain experiments follow a transfer learning paradigm: the model is pre-trained on the source domain and then fine-tuned on a small amount of labeled target-domain data. The goal is to evaluate whether self-supervised pre-training on the source domain can benefit recognition performance on a target domain with limited labeled data.

As illustrated in Figure 3a, when pre-trained on SD5, RD14, and RD11 and fine-tuned on RD13, our method achieves transfer recognition accuracy above 95% across all three source domains, consistently exceeding the performance of 1D-MAE. Particularly, pre-training on RD11 yields an accuracy of 96.77% for our model, significantly outperforming 1D-MAE and GAE by margins of 13% and 16%, respectively. Figure 3b reports the cross-domain results with RD14 as the target dataset. Although both MAE and SSL-MRA attain accuracy higher than 90%, SSL-MRA surpasses 1D-MAE by roughly 2% to 5% under all source-domain configurations. In addition, based on RD11 pre-training, the accuracy after transfer fine-tuning on RD14 is only 88.65% for GAE. For RD11 as the target domain (Figure 3c), transfer accuracy does not exceed 80% for 1D-MAE when pre-trained on RD13 and RD14. In contrast, our SSL-MRA reaches 86.21% and 83.22%, respectively, delivering clear performance improvements over the baseline. To further improve recognition accuracy across various datasets, we construct a mixed training set combining RD13, RD14, and RD11 for supplementary pre-training, followed by fine-tuning on each individual real-measured dataset. This multi-source pre-training scheme further boosts radar emitter recognition accuracy, with the highest transfer accuracy of 89.52% obtained on RD11. This also offers us an insight: when samples from the target domain are insufficient, incorporating data from other domains for mixed pre-training can, to some extent, enhance the model’s recognition capability within the target domain.

### 3.2. Ablation Study

We conduct ablation experiments to investigate the individual contributions of the two proposed self-supervised pretext losses to downstream radar emitter recognition accuracy. Four experimental setups are designed for comparison:

(1) Scratch: The SSL-MRA encoder architecture (ViT-based) trained entirely from scratch without pre-trained weights or any self-supervised pretext tasks.
(2) w/o MPA: Pre-training is performed while removing the Masked Prediction Alignment (MPA) loss.
(3) w/o MDA: Pre-training is performed while removing the Masked Discrete Alignment (MDA) loss.
(4) with all: Full pre-training equipped with both MPA and MDA losses.

Detailed quantitative results are summarized in Table 3. When trained from scratch, the model achieves less than 95% accuracy on the simulated dataset SD5 and merely 76.64% on the real measured dataset RD11. By contrast, pre-training with either MPA or MDA alone yields consistent accuracy gains across all datasets. When only MDA is activated, the model obtains 95.85% on RD13 and 93.57% on RD14, with RD11 accuracy rising to 83.00%. When only MPA is adopted, clear performance improvements are also observed on RD13 and RD14; especially on RD11, the accuracy reaches 86.36%, nearly 10 percentage points higher than the scratch training baseline. When MPA and MDA are jointly optimized as dual pretext objectives, the model achieves further accuracy improvements, hitting 99.25% on SD5 and the peak value of 86.68% on RD11. These ablation results verify that the two proposed masked representation alignment losses based on deep latent features are critical for boosting the recognition performance of downstream radar emitter recognition tasks.

### 3.3. Analysis of Mask Ratio and Patch Size

We perform supplementary ablation experiments on three real measured datasets to investigate how mask ratio and patch size affect the final recognition performance, with quantitative results visualized in Figure 4.

Figure 4a reports the recognition accuracy under five different mask ratios: 20%, 30%, 40%, 50%, and 60%. For RD13 and RD14, the model maintains accuracy above 98% and 95%, respectively, when the mask ratio ranges from 40% to 60%, and the peak performance is consistently attained at a mask ratio of 50%. For RD11, the highest accuracy of 86.68% is also achieved with a 50% mask ratio. By comparison, lower mask ratios (20% and 30%) lead to obvious accuracy degradation across all three datasets. Accordingly, we fix the mask ratio to 50% in all subsequent experiments.

With the mask ratio fixed at 50%, we further test patch sizes of 1, 2, 4, 8, and 16 to analyze their influence on recognition capability. As presented in Figure 4b, patch sizes of 2 and 4 enable RD13 to exceed 98% accuracy, where size 4 delivers the optimal performance. For RD14, the maximum accuracy also appears at patch size 4. In contrast, RD11 achieves its best recognition result when the patch size is set to 2. Overall, patch sizes 2 and 4 yield competitive recognition accuracy on all datasets. To strike a balance between recognition accuracy and computational overhead, we adopt 4 as the final patch size throughout our model.

### 3.4. Feature Representation Visualization at Different Training Stages

We adopt a mixed pre-training strategy combining all three real measured datasets and utilize t-SNE [41] to visualize deep feature embeddings output by the Transformer encoder at three training stages: randomly initialized state (without pre-training), post-pre-training, and post-fine-tuning optimized for radar emitter recognition. The hyperparameters of t-SNE are set as follows: n_components = 2, perplexity = 30, n_iter = 1000, init = pca, and random_state = 42. Each row of subplots in Figure 5 sequentially presents embedding results of the initial stage, pre-trained stage, and fully fine-tuned stage.

The visualization reveals that features of distinct radar emitter types are heavily overlapped and indistinguishable under random initialization. After self-supervised pre-training, the encoder yields embeddings with preliminary inter-class separability in the latent space. After downstream fine-tuning, the feature discrimination is further enhanced: inter-cluster distances between different radar categories are enlarged, while intra-class feature distributions become tighter and more compact. Overall, the t-SNE results indicate, to some extent, that the proposed self-supervised method can distinguish between different radar emitters in the high deep feature space, and that this discriminative capability is further enhanced following supervised fine-tuning. Subsequent task-specific fine-tuning further refines discriminative latent representations, laying a solid foundation for accurate radar emitter recognition.

### 3.5. Robustness Analysis Under Different Complex EM Environments and Different Radar Frequency Bands

To further validate the robustness of the proposed method across varying complex electromagnetic environments and different levels of data sample distinctiveness, we calculated the recognition accuracy using the SD5 dataset under various pulse spurious rates and loss rates, as shown in Table 4. In scenarios involving only pulse loss, the recognition rate dropped from 99.85% to 98.60% as the loss rate increased from 10% to 50%. Similarly, in scenarios involving only false noise, the recognition rate fell by 1.3% as the pulse spurious rate rose from 5% to 25%. Although the recognition rate declined more significantly when both spurious pulses and pulse loss are present, it remained above 98% even under the worst case conditions, which demonstrates the proposed method’s robustness and adaptability in complex EM environments.

In addition, we analyzed the discriminability of radar types within each frequency band for the RD11 and RD13 datasets. As shown in Figure 6, confusion primarily occurs within the S-band and C-band radar groups, and categories 1, 3, 4, 7, and 8 correspond to S-band radars, whereas categories 0, 2, 4, 5, and 9 correspond to C-band radars. Within the C-band, the two radar types that are most difficult to distinguish are categories 4 and 9, and this is because type 9 is an improved version of type 4, resulting in very similar RFs and PRIs with significant overlap. For RD13’s confusion matrix in Figure 7, classification confusion primarily arises from the three S-band categories—3, 5, and 11—and yet the maximum confusion value is only 0.02. Therefore, the proposed method demonstrates high performance in the radar emitter recognition task on the RD13 dataset. The above results demonstrate the robustness of the proposed method for radar recognition across different measured datasets and frequency bands.

### 3.6. Linear Probing Capabilities of Masked Self-Supervised Models

We conducted linear probing on three types of mask-based self-supervised learning methods: 1D-MAE, GAE, and our proposed SSL-MRA. And we present their accuracy and F1 scores across four datasets in the Table 5. The experimental results show that all three methods achieved over 90% accuracy on the simulated dataset SD5. Among the three real measured datasets, the baseline 1D-MAE performed the worst, whereas GAE and our method (which both employ a contrastive learning strategy) perform better. Notably, our method achieved over 90% recognition accuracy on the shipborne radar dataset RD13 and the ground-based radar dataset RD14, and it exceeded 80% even on the most challenging dataset, RD11.

### 3.7. Comparison of Time Cost

We compared the pre-training time of several self-supervised models across all experimental datasets. As shown in Table 6, the time cost is primarily determined by the data volume and the number of model parameters, all methods incurred the highest time costs on the RD5 dataset, while the pre-training time per epoch is lowest on the RD11 dataset, our method taking only 2.3 s. The GAE and 1D-MAE models both have parameter counts exceeding 85 million. However, because the GAE involves multiple encoder computations, its time cost is higher than that of the standard 1D-MAE. Although the MoCo has a parameter count similar to ours, it requires separate computations for both positive and negative samples, resulting in a higher time cost than our method.

Furthermore, the inference time on the radar emitter recognition task with a fixed batch size of 512 is shown in Table 7. Since only a single encoder component is used for feature extraction without a decoder or the calculation of additional losses, the inference times for Transformer, MoCo, and our method are all under 0.2 s. Due to the high dimensionality and large number of parameters in the encoders for 1D-MAE and GAE, their inference time is relatively long, exceeding 0.4 s for the same volume of data.

### 3.8. Impact of Original Pulse Sequence Length on Recognition Accuracy

Given the wide range of pulse sequence lengths in the RD11 dataset and its generally poor overall recognition accuracy, we conducted further analysis based on this dataset. We divided the fine-tuning data into five subsets categorized by sequence length: less than 32; between 32 and 64; between 64 and 128; between 128 and 256; and greater than 256. We then analyzed the trend of accuracy relative to the original sequence length and presented the analysis and the results as shown in Table 8. It is evident that as the pulse sequence length increases, the recognition accuracy for both full fine-tuning and linear probing improves. Notably, when the length is less than 64, the fine-tuning accuracy is poor, falling far below the average accuracy of 82.68% listed in Table 2. And it drops to just 71.43% when the length is less than 32. Thus, longer pulse sequences are more conducive to radar emitter recognition. However, since the processing stages (such as reception and sorting) of measured data often result in very short PDW sequences for individual emitters, achieving higher recognition accuracy based on short pulse sequences is a topic worthy of further research.

### 3.9. Impacts of MPA and MDA on Recognition Accuracy Under Different Pulse Spurious Rates and Loss Rates

To investigate the complementary roles of MPA and MDA, we evaluate the model with each loss removed separately under pulse loss and spurious pulses at rates of 10–40% as shown in Table 9. Under pulse loss, removing MDA causes a clear degradation, with accuracy decreasing from 98.96% to 97.68% and F1 remaining at a low level of 53.62–58.67% as the loss rate increases. In contrast, removing MPA maintains high performance across all loss rates (accuracy ≥ 99.10%, F1 ≥ 95.14%), indicating that MDA is the dominant mechanism for compensating pulse loss. This is because pulse loss destroys the sequence structure and token context, whereas the discrete semantic alignment of MDA preserves consistent token assignments despite missing pulses. Under spurious pulses, removing MDA leads to the largest performance drop (accuracy from 91.90% to 87.30%), while removing MPA also reduces accuracy (from 99.20% to 95.90%), suggesting that MDA plays the primary role and MPA provides complementary robustness against structural contamination. For measurement noise, which is a relatively small perturbation, the model inherently denoises through self-supervised pre-training on noisy real-world sequences, and MPA further smooths minor feature drift, enabling the model to adapt to such variations without a dedicated denoising module. Overall, these results demonstrate that MPA and MDA operate at different granularities of the feature space: MDA anchors discrete semantic consistency against structural perturbations (pulse loss and spurious pulses), while MPA maintains continuous feature smoothness against numerical perturbations (measurement noise), and their combination yields complementary robustness.

## 4. Discussion

Comprehensive experiments verify the effectiveness of the proposed masked representation alignment-based SSL-MRA for radar emitter recognition. Our core hypothesis is that joint Masked Prediction Alignment (MPA) and vector-quantized Masked Discrete Alignment (MDA) extract more robust PDW semantic features than conventional SSL models like MoCo, 1D-MAE, and GAE. Source-domain results strongly support this claim: SSL-MRA achieves highest average accuracy on simulated and real measured datasets against mainstream supervised and self-supervised baselines. Most existing radar SSL methods rely on a single pretext task such as autoregressive prediction or simple reconstruction, which easily overfits dataset-specific noise and fails to separate radar category features from interference. By contrast, our dual losses jointly regularize temporal consistency and discrete semantic clustering, bringing consistent accuracy gains across all four datasets. This aligns with prior research showing multi-objective SSL improves discriminability for multi-parameter time series. Cross-domain transfer experiments confirm the dual-branch momentum update and discrete codebooks learn domain-invariant features to mitigate simulation-real distribution shifts. Traditional single-encoder MAE variants suffer severe generalization degradation on cross domain measured data. Our EMA-updated momentum encoder stabilizes latent prototypes, while discrete token mapping suppresses domain-specific noise. SSL-MRA consistently outperforms 1D-MAE by a large margin across all transfer settings, consistent with contrastive learning findings that momentum branches boost cross-domain generalization.

Notably, performance degrades on RD11, as fixed-length cropping removes its unique long-range pulse patterns. Most prior PDW recognition works ignore this information loss when unifying sequence lengths. Ablation results prove MPA and MDA are mutually complementary; removing either loss weakens inter-class feature separation, while their joint optimization fills the gap of missing multi-dimensional alignment constraints in existing PDW SSL methods. Models trained from scratch perform far worse, confirming SSL pre-training unlocks valuable information from unlabeled radar pulses. Hyperparameter ablation justifies our 50% mask ratio and patch size 4, balancing sufficient masked supervision and complete local multi-parameter coupling, consistent with optimal settings reported in the masked time-series modeling literature.

However, our work has several limitations to address in future work. First, fixed-length preprocessing truncates long-range temporal dependencies; we will design adaptive variable-length patch segmentation to retain the full pulse context. Second, static EMA codebook updates cannot handle imbalanced radar categories, so dynamic clustering strategies will be explored as alternatives.

## 5. Conclusions

This paper proposes a masked representation alignment-based self-supervised learning framework (SSL-MRA) to extract discriminative feature representations and realize accurate radar emitter recognition based on multi-parameter PDW sequences. The proposed SSL-MRA architecture is constructed with a dual-branch Transformer encoder, a cross-attention masked predictor and discrete tokenizer, which jointly realizes two mutually complementary self-supervised pretext tasks, namely, masked prediction alignment (MPA) and masked discrete alignment (MDA). The MPA task enforces continuous feature consistency between masked tokens and predicted latent embeddings, while the MDA task leverages vector quantization to build discrete semantic supervision signals, which jointly drive the model to capture robust multi-parameter coupling patterns hidden in noisy PDW time series. Extensive experiments conducted on one simulated dataset and three real measured radar datasets comprehensively verify the superiority of SSL-MRA. Compared with classic supervised models (ResNet-50, vanilla Transformer) and mainstream self-supervised time-series baselines (MoCo, TimeDART, 1D-MAE, GAE), our method achieves the highest average recognition accuracy on source-domain tasks. Meanwhile, cross-domain transfer experiments demonstrate that SSL-MRA possesses stronger domain generalization capability than conventional MAE-based pre-training methods, effectively alleviating the severe distribution mismatch between simulated training samples and real measured radar data.

Nevertheless, the current preprocessing pipeline relies on fixed-length cropping to unify the length of input PDW sequences, which inevitably truncates long-range temporal pulse correlation information and causes irreversible feature loss. Therefore, designing flexible adaptive encoding and segmentation strategies to preserve the complete temporal context of original variable-length PDW sequences is expected to further improve the feature extraction and generalization performance of the pre-trained model.

## Figures and Tables

**Figure 1:**
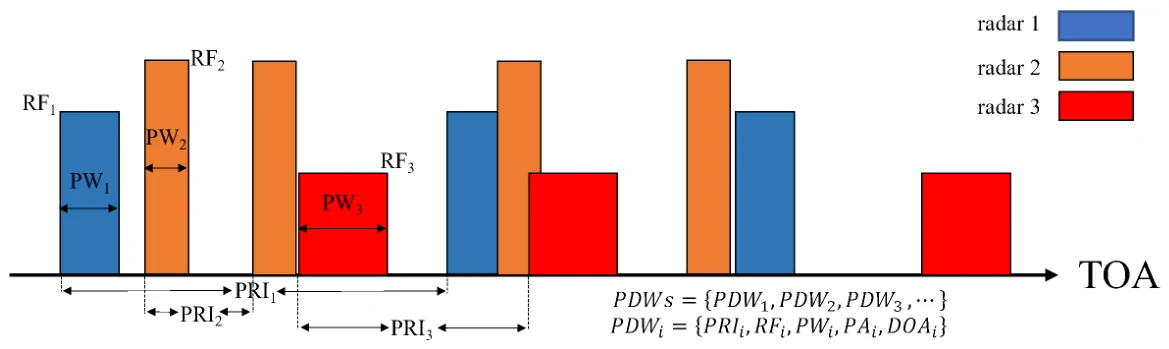
The diagram of radar pulse description words (PDWs). The PDW of each intercepted pulse is a multi-parameter sequence, which comprises the time of arrival (TOA), pulse width (PW), radio frequency (RF), pulse amplitude (PA), and direction of arrival (DOA). Typically, we convert TOA into pulse repetition interval (PRI) using first-order differencing.

**Figure 2:**
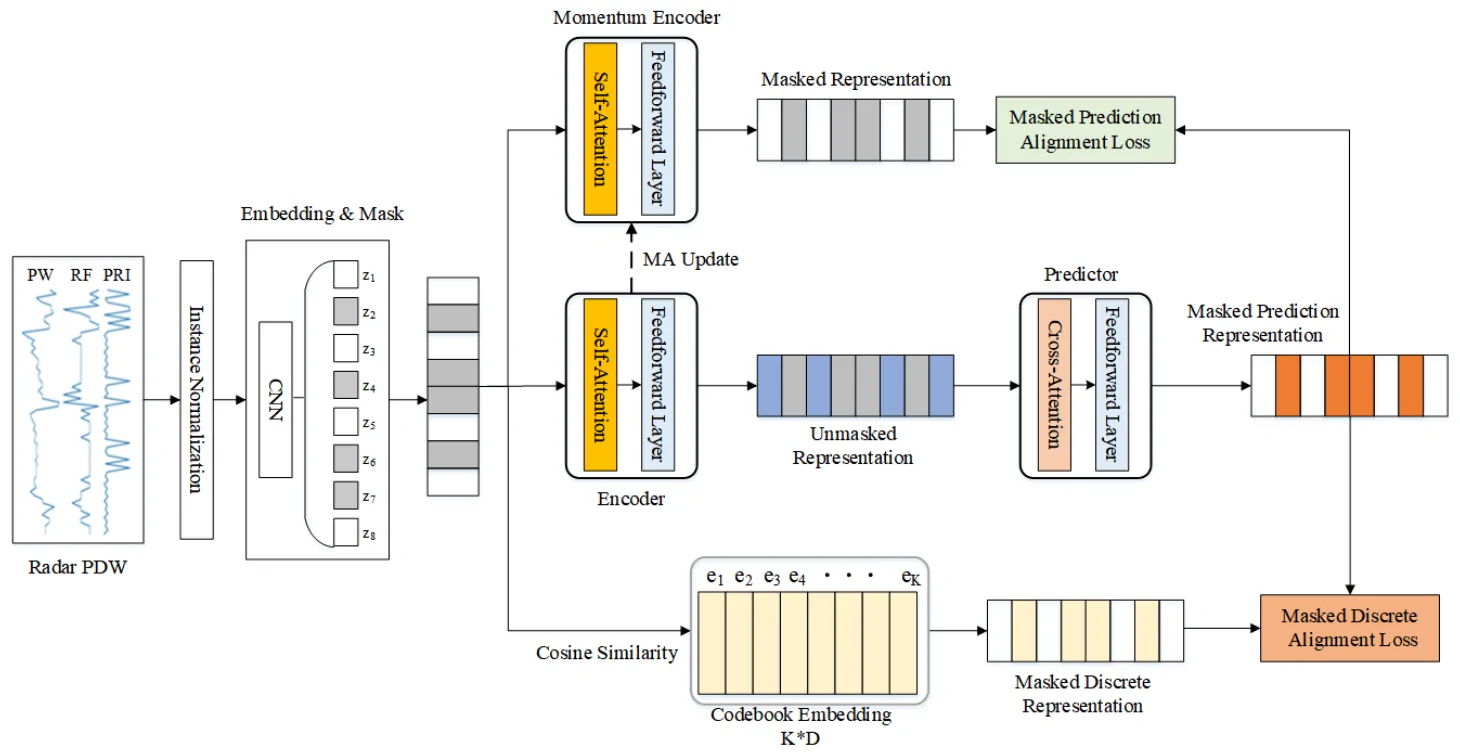
Overall architecture of the proposed masked representation alignment-based self-supervised learning method for radar emitter recognition. The framework consists of four core modules: a preprocessing and embedding module, a dual-branch contextual feature representation module, a masked prediction representation module, and a masked discrete representation module.

**Figure 3:**
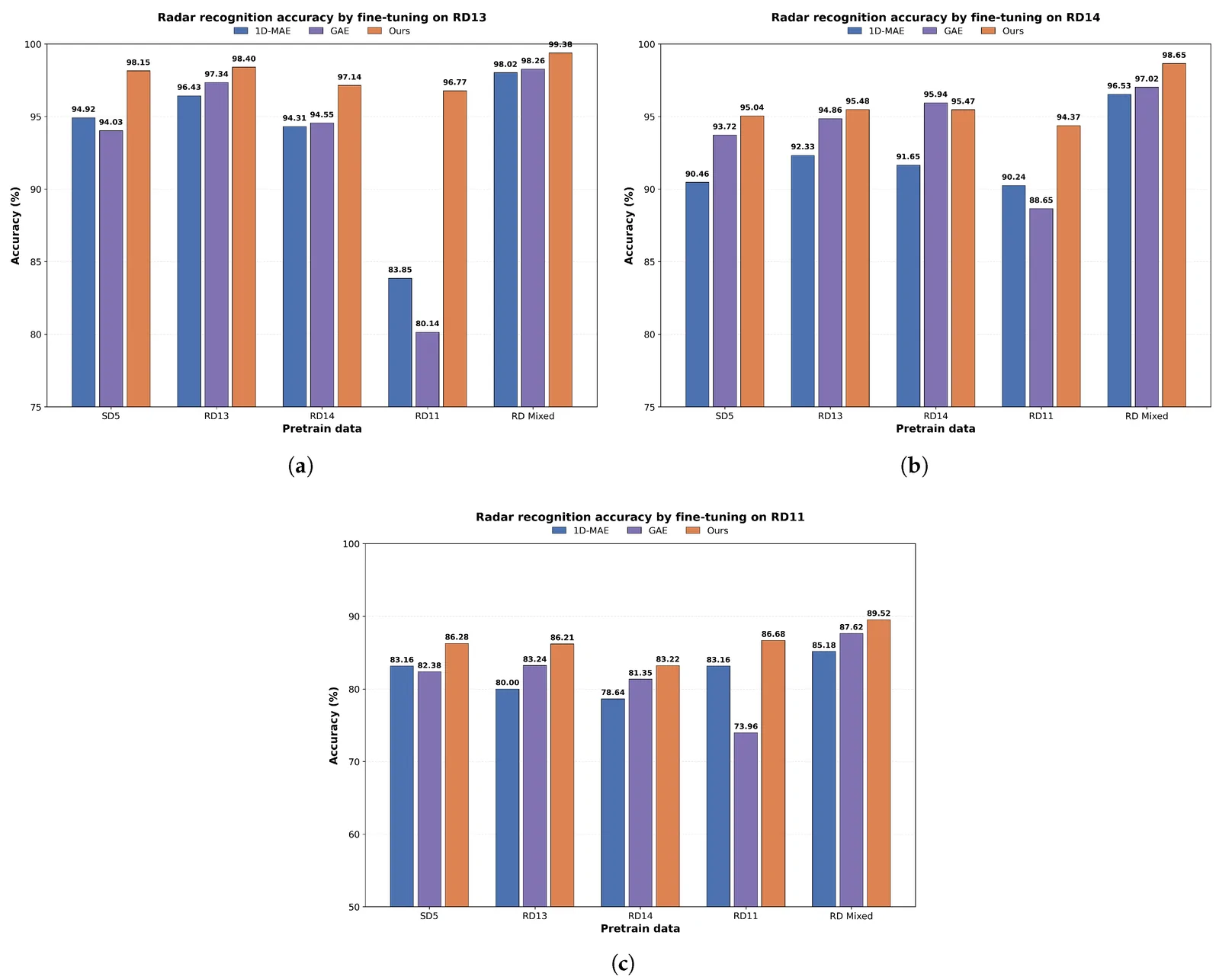
Comparison of cross-domain generalization accuracy (%). (**a**) Recognition accuracy with RD13 as the target domain. (**b**) Recognition accuracy with RD14 as the target domain. (**c**) Recognition accuracy with RD11 as the target domain.

**Figure 4:**
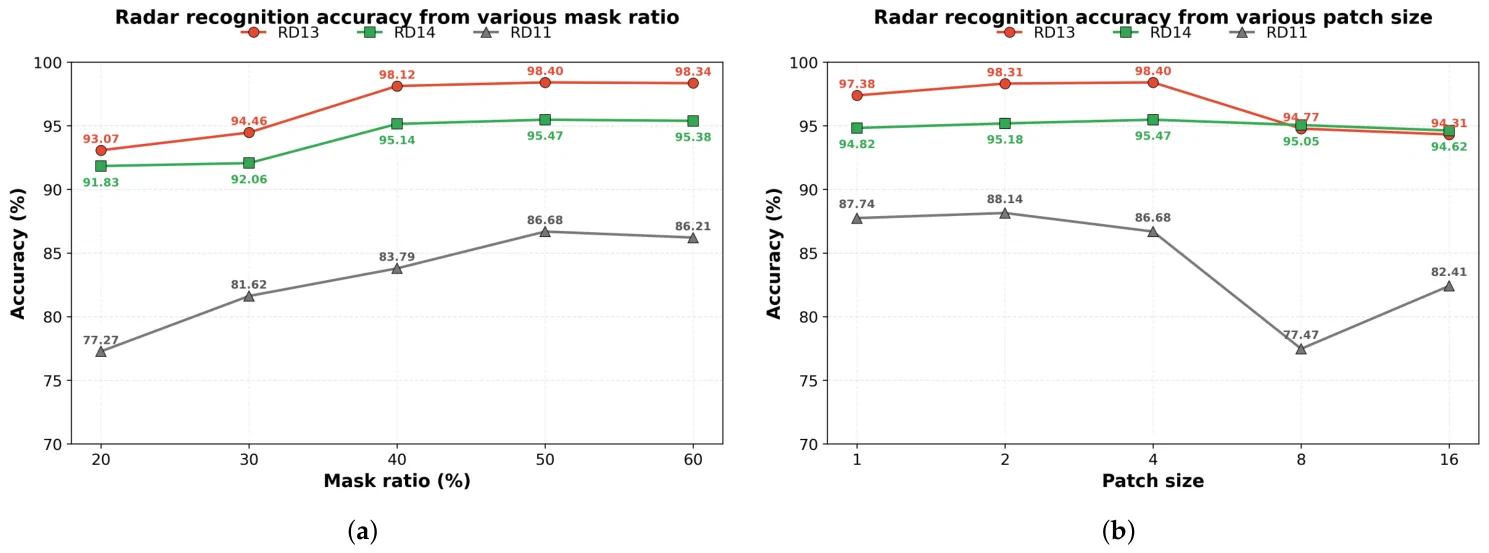
Recognition accuracy under different mask ratios and patch sizes (%).

**Figure 5:**
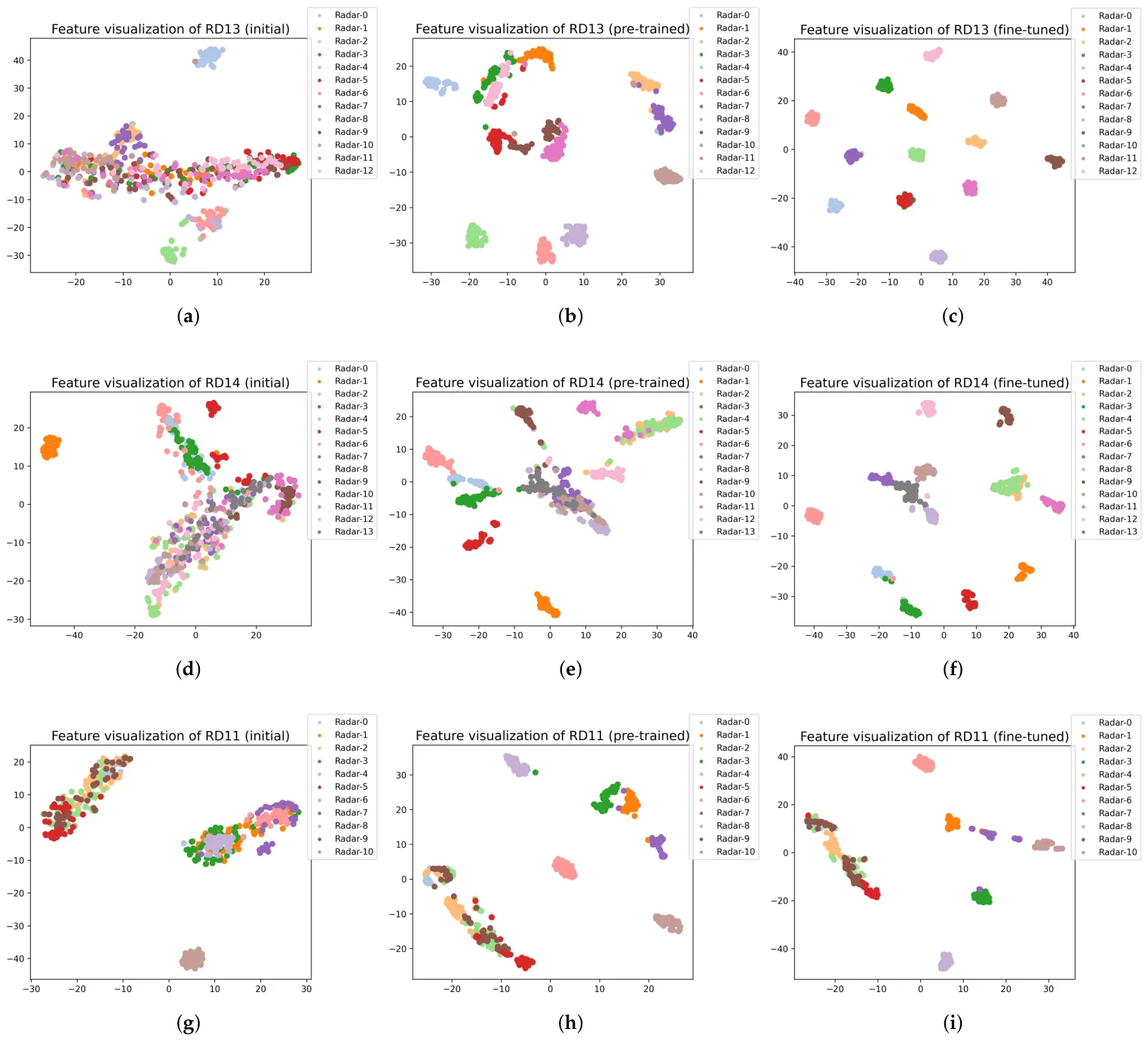
t-SNE visualization of deep feature embeddings extracted from PDW sequences on three real measured datasets. (**a**–**c**) Correspond to RD13 features at three training stages: initialization, post-pre-training, and post-fine-tuning; (**d**–**f**) illustrate RD14 feature distributions; (**g**–**i**) display RD11 feature distributions.

**Figure 6:**
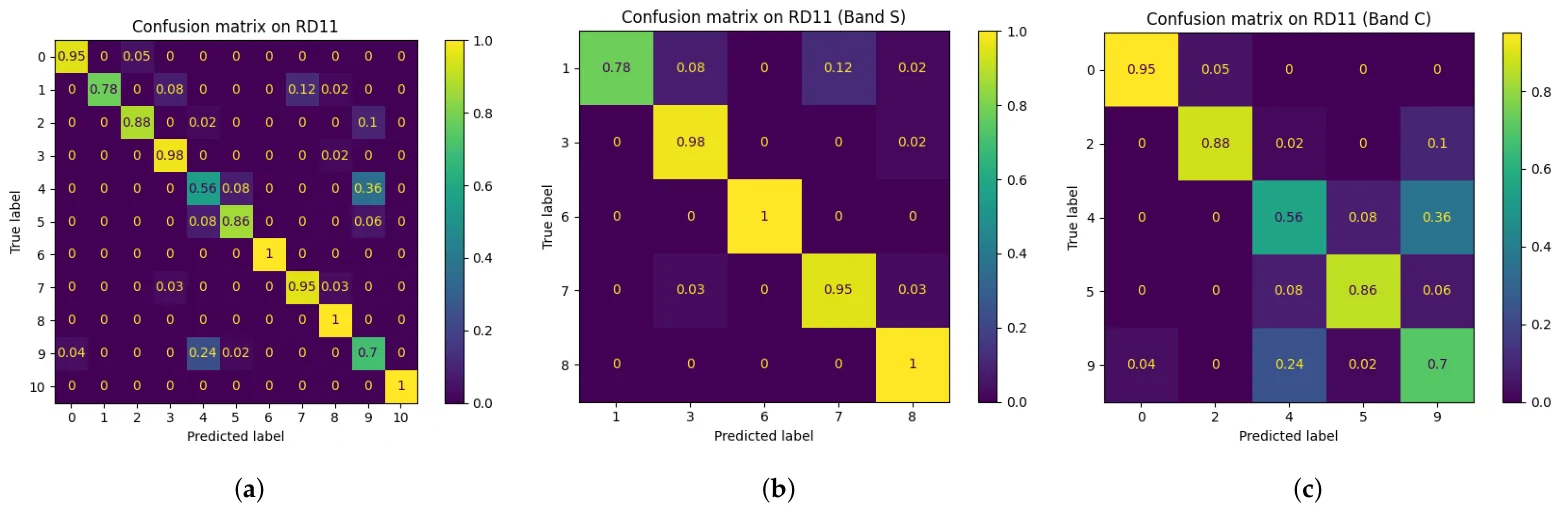
Confusion matrix for RD11. (**a**) Overall confusion matrix for all radar categories; (**b**) confusion matrix for S-band radar categories; (**c**) confusion matrix for C-band radar categories.

**Figure 7:**
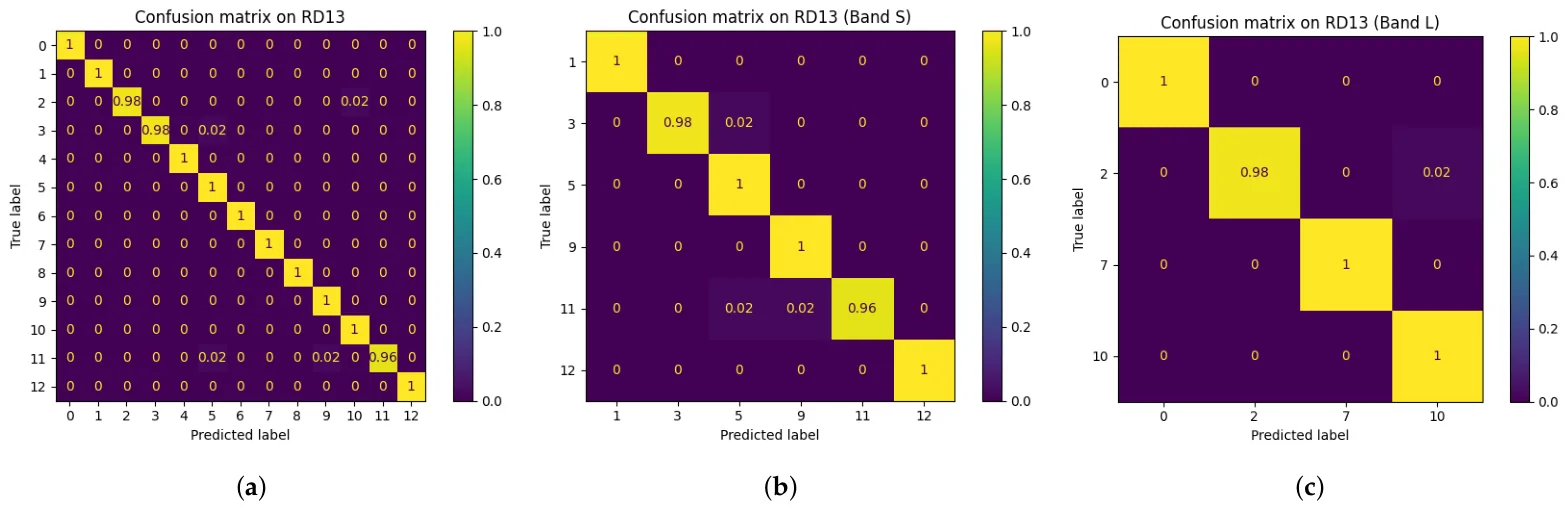
Confusion matrix for RD13. (**a**) Overall confusion matrix for all radar categories; (**b**) Confusion matrix for S-band radar categories; (**c**) Confusion matrix for L-band radar categories.

**Table 1:** Dataset partitioning.

Dataset	Pre-Train	Fine-Tune (Train)	Fine-Tune (Test)	Number of Categories
SD5	80,000	16,000	4000	5
RD13	20,394	2600	650	13
RD14	16,299	2800	700	14
RD11	9819	2200	550	11

**Table 2:** Comparison of source-domain recognition accuracy with baseline methods (mean ± std, %).

Method	SD5	RD13	RD14	RD11	Average
Resnet-50	82.28 ± 0.71	80.15 ± 0.55	79.62 ± 0.72	82.29 ± 0.84	81.09 ± 0.71
Transformer	91.32 ± 0.32	83.21 ± 0.38	82.13 ± 0.38	83.35 ± 0.53	85.02 ± 0.40
TimeDART	90.05 ± 0.86	77.84 ± 0.90	76.16 ± 0.88	75.25 ± 0.85	79.83 ± 0.87
MoCo	93.36 ± 0.62	90.42 ± 0.65	87.25 ± 0.68	81.74 ± 0.67	88.19 ± 0.66
1D-MAE	98.22 ± 0.45	96.43 ± 0.47	91.65 ± 0.49	83.16 ± 0.51	92.37 ± 0.48
GAE	96.09 ± 0.28	97.34 ± 0.32	**95.94 ± 0.32**	73.96 ± 0.37	90.83 ± 0.32
SSL-MRA (Ours)	**99.25 ± 0.19**	**98.40 ± 0.27**	95.47 ± 0.29	**86.68 ± 0.35**	**94.95 ± 0.28**

Bold values denote the best performance per column.

**Table 3:** Radar emitter recognition accuracy under different pretext task combination (%).

Dataset	Scratch	w/o MPA	w/o MDA	with All
SD5	91.74	96.24	96.43	99.25
RD13	85.35	95.85	93.07	98.40
RD14	84.02	93.57	92.96	95.47
RD11	76.64	83.00	86.36	86.68

**Table 4:** Radar emitter recognition accuracy under different pulse spurious rates and loss rates (%).

Condition	10/5	20/10	30/15	40/20	50/25
Loss	99.85	99.68	99.42	98.80	98.60
Spurious	99.80	99.59	99.31	98.70	98.50
Loss & Spurious	99.75	99.52	99.22	98.64	98.35

The notation “A/B” denotes spurious rate *A* (%) and pulse loss rate *B* (%).

**Table 5:** Linear probing accuracy of masked self-supervised models (Accuracy/F1 Score, %).

Method	SD5	RD13	RD14	RD11	Average
	Acc/F1	Acc/F1	Acc/F1	Acc/F1	Acc/F1
1D-MAE	90.14/90.02	87.52/87.31	60.95/60.80	68.04/67.85	76.66/76.50
GAE	93.82/93.24	95.27/94.68	87.62/87.35	71.63/71.20	87.09/86.62
Proposed	93.25/93.02	95.38/95.23	93.00/92.96	82.81/82.69	91.11/90.98

**Table 6:** Pre-train time cost of different SSL model.

Method	SD5	RD13	RD14	RD11	Average
TimeDART	26.4 s/epoch	7.5 s/epoch	6.2 s/epoch	2.5 s/epoch	10.7 s/epoch
MoCo	35.3 s/epoch	8.4 s/epoch	7.5 s/epoch	3.7 s/epoch	13.7 s/epoch
1D-MAE	45.1 s/epoch	10.4 s/epoch	9.2 s/epoch	4.1 s/epoch	17.2 s/epoch
GAE	58.4 s/epoch	14.5 s/epoch	12.2 s/epoch	5.2 s/epoch	22.6 s/epoch
Ours	25.2 s/epoch	7.2 s/epoch	5.0 s/epoch	2.3 s/epoch	9.9 s/epoch

The notation “s/epoch” denotes the seconds required to train each epoch.

**Table 7:** Inference time of different recognition model.

Method	Resnet-50	Transformer	TimeDART	MoCo	1D-MAE	GAE	Ours
Time	0.24 s	0.15 s	0.28 s	0.16 s	0.42 s	0.45 s	0.17 s

**Table 8:** Recognition accuracy under different original sequence lengths (Accuracy/F1 Score, %).

Method	Original Sequence Length				
	≤32	(32, 64]	(64, 128]	128, 256]	≥256
	Acc/F1	Acc/F1	Acc/F1	Acc/F1	Acc/F1
Fine-tune	71.43/66.67	82.29/82.46	86.67/90.01	91.58/85.62	98.57/85.19
Linear probing	78.57/70.95	78.65/80.72	82.96/87.60	85.26/76.83	95.71/86.28

**Table 9:** Recognition performance w/o MPA or MDA under different distortion rates (Acc/F1, %).

Method	Distortion	Distortion Rate			
		10%	20%	30%	40%
w/o MPA	lost	99.10/97.87	99.35/97.73	99.44/97.07	99.27/95.14
	spurious	99.20/92.30	97.70/91.80	96.10/89.30	95.90/89.80
w/o MDA	lost	98.96/53.62	98.61/55.73	98.15/57.44	97.68/58.67
	spurious	91.90/52.00	90.60/52.60	89.10/54.50	87.30/52.30

## Data Availability

The dataset SD5 consists of simulated data, which we can upload after the paper has been accepted. However, the datasets of RD13, RD14, and RD11 are unavailable due to privacy restrictions; they cannot be shared, as these real measured datasets originate from restricted or defense-related sources. We ask for your understanding in this matter.

## Abbreviations

The following abbreviations are used in this manuscript:

SSL	Self-supervised learning
PDW	Pulse description word
ESM	Electronic support measures
MRA	Masked representation alignment
PRI	Pulse repetition interval
RF	Radio frequency
PW	Pulse width
NLP	Natural language processing
CV	Computer vision
CL	Contrastive learning
STE	Straight through estimator
MAE	Masked autoencoder
VQ	Vector quantization
EMA	Exponential moving average
MPA	Masked prediction alignment
MDA	Masked discrete alignment
